# From phenotypical investigation to RNA-sequencing for gene expression analysis: A workflow for single and pooled rare cells

**DOI:** 10.3389/fgene.2022.1012191

**Published:** 2022-11-14

**Authors:** Tania Rossi, Davide Angeli, Giovanni Martinelli, Francesco Fabbri, Giulia Gallerani

**Affiliations:** ^1^ Biosciences Laboratory, IRCCS Istituto Romagnolo per lo Studio dei Tumori (IRST) “Dino Amadori”, Meldola, Italy; ^2^ Unit of Biostatistics and Clinical Trials, IRCCS Istituto Romagnolo per lo Studio dei Tumori (IRST) “Dino Amadori”, Meldola, Italy; ^3^ Scientific Directorate, IRCCS Istituto Romagnolo per lo Studio dei Tumori (IRST) “Dino Amadori”, Meldola, Italy

**Keywords:** RNA-sequencing, NGS, single cell analysis, rare cells, deparray, gene expression, circulating tumor cells

## Abstract

Combining phenotypical and molecular characterization of rare cells is challenging due to their scarcity and difficult handling. In oncology, circulating tumor cells (CTCs) are considered among the most important rare cell populations. Their phenotypic and molecular characterization is necessary to define the molecular mechanisms underlying their metastatic potential. Several approaches that require cell fixation make difficult downstream molecular investigations on RNA. Conversely, the DEPArray technology allows phenotypic analysis and handling of both fixed and unfixed cells, enabling a wider range of applications. Here, we describe an experimental workflow that allows the transcriptomic investigation of single and pooled OE33 cells undergone to DEPArray analysis and recovery. In addition, cells were tested at different conditions (unfixed, CellSearch fixative (CSF)- and ethanol (EtOH)-fixed cells). In a forward-looking perspective, this workflow will pave the way for novel strategies to characterize gene expression profiles of rare cells, both single-cell and low-resolution input.

## Introduction

In recent years, advances in the development of low-input RNA-sequencing protocols have enabled the characterization of rare cells (Jindal et al., 2018; Nguyen et al., 2018; Rossi and Zamarchi, 2019; Negishi et al., 2022). Circulating tumor cells (CTCs) represent a typical case in point of rare cells in cancer (Yang et al., 2019; Rossi et al., 2021a), and are often investigated through the gold standard enumeration method CellSearch after fixation for prognostic purposes. Another frequently used approach, consisting in the DEPArray platform, allows for the immunophenotypic analysis and handling of single fixed or unfixed cells by exploiting the dielectrophoretic principle. However, experimental workflows for transcriptomic analyses of CTCs analysed using both CellSearch and DEPArray are still lacking.

Herein, we describe an experimental workflow that allows for the analysis, from phenotypical to transcriptomic, of single cells and 10-cell pools. Our findings are pioneering and pave the way to new applications in the study of rare cells like CTCs.

## Materials and methods

### Study design

In this study, we tested the ability of the QIAseq UPX 3’ Transcriptome kit (QIAGEN, Germantown, MD, United States) to provide valuable gene expression data on cells isolated using the DEPArray NxT platform (Menarini Silicon Biosystems, Castel Maggiore, Italy). In particular, samples were tested based on (i) condition (CellSearch fixative (CSF) and ethanol (EtOH)-fixed cells, unfixed cells) and (ii) sample type (single cell, 10-cells pools, RNA).

### Cell culture and fixation

OE33 commercial cells were cultured in RPMI 1640 + 2 mM Glutamine + 10% Foetal Bovine Serum (FBS; Gibco; Thermo Fisher Scientific, Inc., Waltham, MA, United States). For sample preparation, OE33 cells were dissociated through trypsinization, then cellular pellet was split in two for CSF and EtOH fixation, respectively.

CSF fixation was performed using the fixative contained in CellSave Preservative tubes. More specifically, fixation was performed with 100 µl of preservative diluted 1:10 in PBS1X at room temperature. EtOH fixation was performed by adding 200 µl of ice-cold PBS1X and 800 µl of ice-cold EtOH dropwise. Fixation was carried out on ice for 15 min. Successively, each fixed sample was washed with ice cold PBS1X and resuspended with 900 µl of ice-cold PBS1X, then split as follows: (i) 800 µl into a 1,5 ml tube, centrifugated and supernatant discarded and immediately stored at −80°C (for RNA extraction) and (ii) 100 µl into a 1,5 ml tube, centrifugated and supernatant discarded (for DEPArray analysis).

### RNA extraction from cellular pellet

We performed RNA extraction from cellular pellets (CSF and EtOH fixed, unfixed) using the RNeasy Mini Kit (QIAGEN, Germantown, MD, United States) following the instruction provided by the manufacturer, including a DNA digestion step with DNAse. RNA was quantified by Spectrophotometer Nanodrop-ND-1000 (Thermo Fisher Scientific, Waltham, MA, United States) and stored at −80°C until downstream analysis.

### DEPArray analysis

CSF- and EtOH-fixed samples for DEPArray analysis were stained with EpCAM-PE 1:10 (clone HEA-125; Miltenyi Biotech, Bergisch Gladbach, Germany), CKs-PE 1:10 (clone C11; Aczon, Bologna, Italy), cMET-APC 1:10 (clone 95106; R&D Systems, McKinley Pl NE, MN, United States) antibodies and Hoechst 33342 (1 µg/ml; Life Technologies, Carlsbad, CA, United States) for nuclear staining. Samples were resuspended in DEPArray™ buffer for fixed cells (Menarini Silicon Biosystems) for DEPArray analysis and sorting. For unfixed OE33 cells, samples were stained only with cell surface antigen EpCAM-PE 1:10 (clone HEA-125; Miltenyi Biotech) and SYTO-16 Green Fluorescent Nucleic Acid Stain (10 nM; Invitrogen, Waltham, MA, United States) and resuspended in RPMI 1640 medium (Gibco) + 10% FBS (Gibco). At this phase, we assessed staining quality and routability, intended as the percentage of the number of cells routable within the DEPArray cartridge compared to the number of identified cells. To this purpose, the DEPArray instrument at the opening of the CellBrowser™ automatically displays a subpopulation of cells trapped in the cages of the cartridge, thus suitable for routing (routable cells) according to the instrument’s manual. By contrast, un-routable events may include spurious events, cell debris, small cell fragments and cells with impaired cell membrane integrity. For downstream analyses, we isolated sixteen single cells and sixteen 10-cell pools for CSF and EtOH samples, and eight single cells and eight 10-cell pools for the unfixed sample. After recovery, each sample was subjected to PBS1X washing under a sterile hood, and volume reduction (to approximatively ∼2 µl) was performed automatically using VR NxT (Menarini Silicon Biosystems). Samples were immediately stored at −80°C until library preparation.

### Library preparation and sequencing

Libraries were prepared using the QIAseq 3’ UPX Transcriptome kit (96-M, QIAGEN) starting from the above-mentioned single cells and 10-cell pools isolated using DEPArray, and 8 replicates of each RNA sample extracted from fixed (CSF and EtOH) and unfixed OE33 cell pellet. In addition, we included in library preparation the Xpress Ref Universal Total RNA (QIAGEN) (8 replicates) as a reference sample. Cell lysis was carried out directly on the 0,2 ml tube used for DEPArray recovery and under a sterile hood to avoid contaminations. Reactions were kept at room temperature for 30 min, and immediately stored at −80°C for 48 h after lysis. During the reverse transcription step, each sample was assigned a different Cell-ID for downstream demultiplexing and each RNA molecule was tagged with a unique molecular index (UMI), allowing to combine together all individually tagged cDNAs. Finally, libraries were checked for quality using the Agilent High Sensitivity DNA kit (Agilent, Waldbronn, Germany), while concentration was assessed using the QIAseq Library Quantification Assay kit (QIAGEN). After calculation of pM concentration, equimolar pools were prepared, denatured at 1 nM and loaded in a V3-150 cycles cartridge at 3pM concentration. Sequencing 100 × 27 was performed on MiSeq Sequencing system (Illumina Inc., San Diego, CA, United States) including custom primer for Read 2 provided with the kit, following the instructions of the manufacturer.

### Bioinformatic analyses

In this study, bioinformatic and statistical analyses were conducted using the toolkit for NGS data CLC Genomics Workbench version 22.0.1 (QIAGEN), Biomedical analysis plugin. Briefly, raw fastq data generated by the MiSeq instrument were uploaded, and demultiplexing of Cell-IDs was carried out using the function “Analyze QIAseq Samples”. Demultiplexed reads were analyzed using the ready-to-use workflow “Quantify QIAseq UPX 3’”. In this step, reads are trimmed and mapped to targets to quantify gene expression based on merging and then counting UMIs. For differential gene expression analysis, gene expression tracks were prepared by comparing the transcriptomic profile of each sample to the reference XpressRef Universal Total RNA. Data were normalized using the global TMM (Trimmed Mean of M-Method) as suggested by the software for whole transcriptome RNA-sequencing. To identify differentially expressed genes, the fold change threshold was set at 1,5, and *p*-values < 0,05 computed using GLM model were considered statistically significant. Shapiro-Wilk test and histograms were prepared using GraphPad Prism 8.0.2.

## Results

### DEPArray analysis and sample preparation for sequencing

**FIGURE 1:**
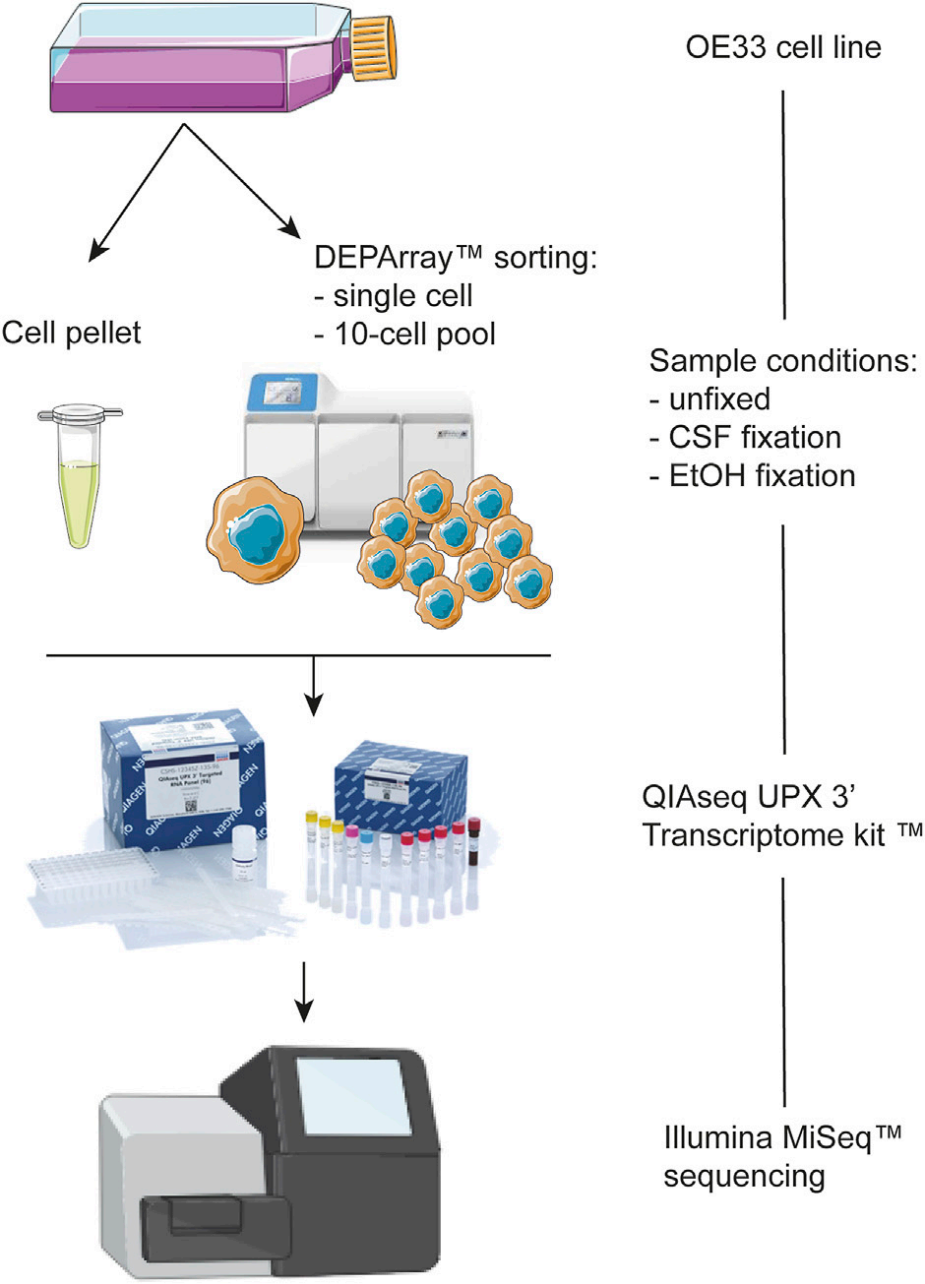
Schematic representation of the workflow of the present study.

In our study, the DEPArray instrument was used for the phenotypic analysis and isolation of fixed (CSF and EtOH) and unfixed OE33 cells as single cells and 10-cell pools (Figure 1).

**FIGURE 2:**
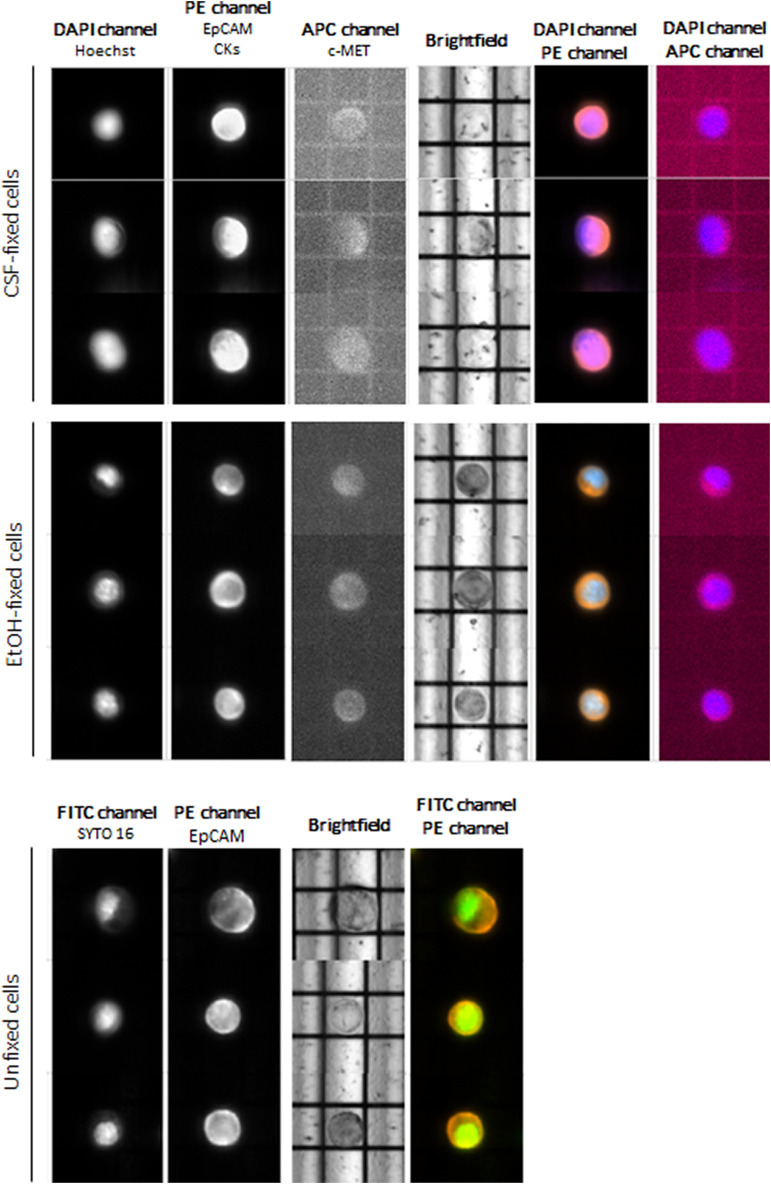
DEPArray images of the most representative OE33 cells. For fixed cells, the DAPI channel was used for nuclear staining using Hoechst 33342, PE channel for epithelial tag [anti-EPCAM and anti-cytokeratins (CKs)], and APC channel for anti-c-MET staining. For unfixed cells, FITC channel was used for nuclear detection (SYTO-16) and PE channel for anti-EpCAM staining.

At first instance, we aimed at evaluating cell routability for each sample we found that EtOH-fixed cells had the highest routability percentage (82%) compared to CSF-fixed cells (72%). Unfixed OE33 cells showed a routability percentage of 80%. Concerning staining, both fixed and unfixed cells had a proper quality, and no false negatives were detected (Figure 2).

### Comparison of mapping efficiency

Firstly, to obtain preliminary information concerning the quality of our data, we investigated mapping efficiency among samples. In Table 1, we report for each sample type and condition the average raw total reads, percentage of reads mapping to genome and to genes and descriptive statistics. To examine the distribution of replicates in each sample we applied the Shapiro-Wilk test, where *p*-values < 0.05 indicates that data significantly deviate from a normal distribution (Shapiro and Wilk, 1965) (Table 1). Due to the data variations among groups, we could not perform further statistical analysis (i.e., statistical comparison).

**TABLE 1:** Report of average total and mapped to genome reads for each sample type and condition.

Samples	Total number of reads					Input reads				
	Minimum	Maximum	Mean	SD	*p-*value	Minimum	Maximum	Mean	SD	*p-*value
Reference	59.477	270.783	153.170,25	76.099,27	**0,375**	51.245	226.290	128.951,50	62.726,14	**0,4078**
RNA OE33 unfixed	248.748	594.665	448.438,5	120.183,56	**0,702**	236.850	565.992	426.478,25	113.806,49	**0,7191**
RNA OE33 CSF-fixed	61.579	215.617	142.828,75	56.189,55	**0,426**	57.171	200.992	132.376,63	52.217,38	**0,4524**
RNA OE33 EtOH-fixed	151.818	303.715	228.154,13	50.547,61	**0,882**	122.842	222.466	167.192,38	33.878,10	**0,7905**
10-cell pool unfixed	289.101	583.312	418.520,63	109.992,91	**0,251**	23.654	52.737	41.184,63	9.949,85	**0,5723**
10-cell pool CSF-fixed	54.841	199.520	106.968,63	45.442,96	**0,118**	4.840	16.120	9.622,69	3.648,66	**0,1179**
10-cell pool EtOH fixed	15.678	102.093	51.776,19	25.466,49	**0,681**	2.972	13.879	7.834,31	3.398,79	**0,5168**
Single cell unfixed	183.002	694.741	365.587,38	155.243,06	**0,186**	12.360	35.924	23.233,13	7.386,15	**0,8692**
Single cell CSF-fixed	12.693	525.242	142.191,75	153.181,30	0,002	1.332	32.209	7.895,13	9.291,71	0,0003
Single cell EtOH-fixed	30.499	149.468	79.477,125	42.770,51	0,024	2.701	14.535	6.590,31	3.460,11	**0,0711**

Sample	Mapped to genome (%)					Mapped to genes (%)				
	Minimum	Maximum	Mean	SD	*p-*value	Minimum	Maximum	Mean	SD	*p-*value
Reference	88,74	92,10	90,43	1,08	**0,99**	95,59	95,99	95,80	0,13	**0,99**
RNA OE33 unfixed	85,97	92,43	88,74	2,46	**0,41**	96,67	97,21	96,90	0,22	**0,17**
RNA OE33 CSF-fixed	81,88	88,60	85,11	2,41	**0,56**	95,12	95,89	95,63	0,27	**0,27**
RNA OE33 EtOH-fixed	87,33	90,94	88,69	1,48	**0,08**	92,14	92,87	92,50	0,28	**0,22**
10-cell pool unfixed	47,14	51,77	49,59	1,56	**0,94**	80,35	87,17	84,36	1,96	**0,31**
10-cell pool CSF-fixed	62,84	72,97	67,64	2,69	**0,99**	84,27	88,64	87,21	1,35	0,04
10-cell pool EtOH fixed	35,74	42,50	39,81	1,93	**0,49**	41,02	49,24	46,53	2,22	**0,10**
Single cell unfixed	31,94	44,09	39,84	4,62	**0,13**	43,34	79,17	68,98	11,71	0,03
Single cell CSF-fixed	24,92	50,84	35,97	7,51	**0,79**	52,42	83,08	69,11	9,16	**0,74**
Single cell EtOH-fixed	32,53	41,99	36,66	2,15	**0,17**	42,97	54,60	50,70	3,01	**0,23**

The number of reads generated by the MiSeq instrument (Total number of reads) and the number of reads after trimming and quality check are reported (Input reads). *p*-value > 0,05 means normal data distribution. CSF: CellSearch Fixative; EtOH: ethanol; SD: standard deviation.

At first instance, we found that the number of generated reads varied among samples. In the EtOH-fixed samples, except for the RNA samples, we obtained the lowest numbers of total reads, probably as a consequence of the effect of EtOH fixation in the qualitative and quantitative assessment of libraries.

The XPress Ref Universal Total RNA, which was used as a reference, displayed the highest average percentage of reads mapped (90,43%). Similar data were observed for RNA samples extracted from unfixed (88,74%) and CSF- and EtOH- fixed (85,11% and 88,69% respectively) OE33 cell pellets. Concerning the mapping efficiency to coding regions, we observed reproducible values among the RNA samples.

In 10-cell pools, the fixation using the CSF fixative appeared more efficient compared to the other conditions. In fact, we found that the CSF-fixed samples showed the highest percentage of average mapped reads (67,64%) compared to unfixed (49,59%) and EtOH-fixed (39,81%) OE33 pools. While the mapping efficiency of reads to coding regions was comparable between unfixed and CSF-fixed 10-cell pools, EtOH-fixed 10-cell pools displayed the worst results (46,53%). By contrast, in all the single cell samples the average percentage of mapped reads, as well as the percentage of reads mapped to coding regions, appeared nearly comparable regardless of the fixation conditions.

Globally, our findings indicate that the application of fixatives does not negatively influence the sequencing quality in terms of reads mapping to the genome in RNA samples, while CSF fixation seems to save a high number of reads by mapping to the genome in 10-cell pool samples. Concerning single cells, mapping efficiency is lower compared to other sample types, regardless of the condition.

### Comparison of features detected

To get an estimation of the genes detected, we investigated the amount of genes mapped by at least one read. To this purpose, we filtered genes having a value greater than 0 in the Total Gene Reads column (the number of reads that are mapped to a gene) from the gene expression track provided by CLC Genomics Workbench (Table 2).

**TABLE 2:** Number of features having > 0 mapping reads for each sample type and condition.

Sample	Replicates	Number of genes with >0 read mapping				
		Minimum	Maximum	Mean	SD	*p-value*
RNA OE33 unfixed	8	11.675	14.276	13.310	852	**0,386**
RNA OE33 CSF-fixed	8	6.673	10.614	9.075	1.421	**0,184**
RNA OE33 EtOH-fixed	8	8.668	10.604	9.593	681,1	**0,559**
10-cell pool unfixed	8	1.214	1.949	1.567	269,3	**0,482**
10-cell pool CSF-fixed	16	538	1562	947,7	341,8	**0,061**
10-cell pool EtOH-fixed	16	160	598	376,5	132,2	**0,658**
Single cell unfixed	8	494	1135	796,1	187,1	**0,806**
Single cell CSF-fixed	16	113	853	343,7	227,9	0,048
Single cell EtOH-fixed	16	169	548	313,9	127,1	0,047

Shapiro-Wilk test was used for normal distribution examination. *p*-value > 0,05 means normal data distribution. CSF: CellSearch Fixative; EtOH: ethanol; SD: standard deviation.

Among the RNA samples, we found that the unfixed sample had the highest average number of features detected, whereas both CSF- and EtOH-fixed RNA samples showed comparable values. However, this discrepancy could be imputable to the higher number of total reads used for the unfixed RNA samples, as previously reported in Table 1.

In the 10-cell pool samples, unfixed samples yielded as expected the higher mean amount of features detected (1.567 genes). At the same time, by comparing the results obtained from fixed samples, while EtOH fixed samples showed the lowest values (mean 376,50 genes) we found a higher number of detected features in 10-cell CSF fixed pools (mean 947,7 genes).

Again, unfixed single cells further demonstrated a higher number of mean features detected (796,1 genes). In fixed samples, our findings highlight a comparable amount of features detected regardless of the fixative used, having Single cell CSF- and EtOH-fixed samples 343,7 and 313,9 genes detected, respectively.

In general, our data highlight that the lack of fixation provides the best results in terms of features detected in all the sample types (RNA, 10-cell pools and single cells). At the same time, while the fixation seems not to impact the quality of the sequencing in terms of detected features in RNA samples, EtOH fixation is the worst in terms of detected features when working with 10-cell pools and single cells.

### Genes detected as expressed

In order to further investigate gene expression, we binned the annotated genes in 5 groups based on their Reads Per Kilobase per Million reads (RPKM): (a) 1 < RPKM ≤ 100, (b) 100 < RPKM ≤ 1.000, (c) 1.000 < RPKM ≤ 10.000 and (d) RPKM > 10.000 (Table 3).

**TABLE 3:** Number of genes detected binned by reads per kilobase per million reads (RPKM).

Sample type and condition	Reads per kilobase per million reads (RPKM)			
	1 < RPKM ≤ 100	100 < RPKM ≤ 1.000	1000 < RPKM ≤ 10.000	RPKM > 10.000
RNA OE33 unfixed	9.143	1.840	90	7
RNA OE33 PF fixed	5.582	1.888	112	2
RNA OE33 EtOH fixed	4.062	1.300	53	8
10-cell pool unfixed	0	541	209	8
10-cell pool PF fixed	0	57	247	14
10-cell pool EtOH fixed	0	0	65	15
Single cell unfixed	0	168	132	24
Single cell PF fixed	0	0	73	20
Single cell EtOH fixed	0	0	39	21

The RPKM data was obtained from the Gene Expression Track generated as output by the CLC Genomics Workbench.

**FIGURE 3:**
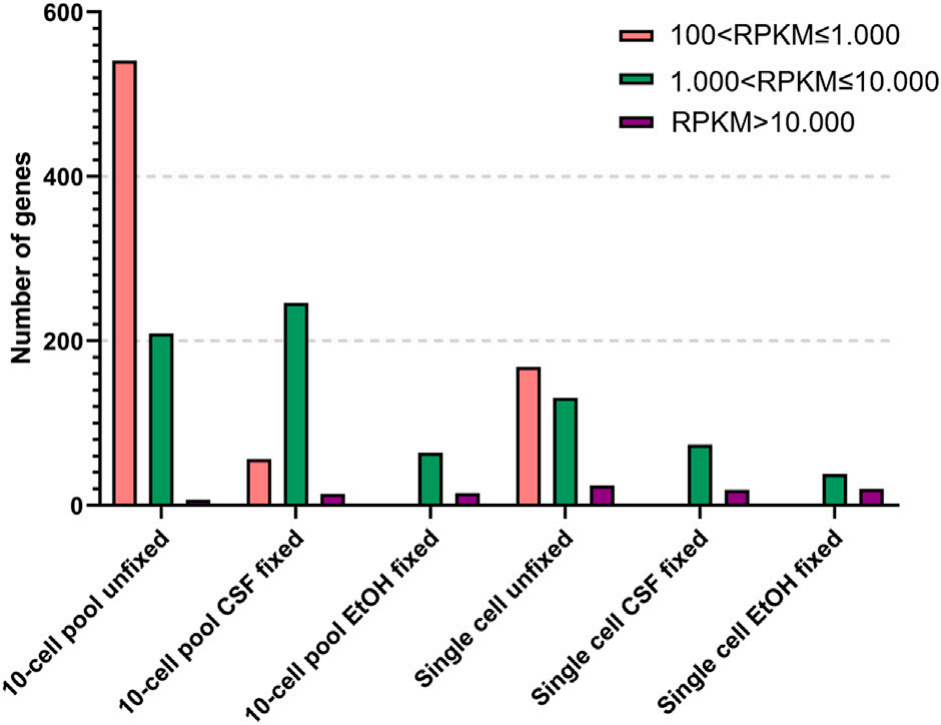
Detected genes binned by reads per kilobase per million reads (RPKM) in 10-cell pools and single cells based on their condition.

As expected, the RNA samples displayed a higher amount of genes expressed at low levels compared to the other samples. However, although the number of genes is comparable, the RNA OE33 EtOH fixed sample tends to present a lower number of genes in each group compared to the CSF-fixed and the unfixed RNA samples. In the 10-cell pools samples, the analysis failed to detect genes with 1 < RPKM≤100, and genes with 100 < RPKM ≤ 1.000 were identified only in unfixed and CSF-fixed samples. Again, the EtOH fixed sample demonstrated a lower efficiency compared to the other samples. Conversely, the unfixed and CSF-fixed sample showed comparable data for genes having 1.000 < RPKM ≤ 10.000 and RPKM > 10.000 (Figure 3).

Finally, in single cell samples genes with 100 < RPKM ≤ 1.000 were found only in unfixed samples, which also yielded the higher amount of genes with 1.000 < RPKM ≤ 10.000 compared to the fixed samples. Simultaneously, we observed that all the samples shared a comparable number of highly expressed genes with RPKM > 10.000.

Globally, these findings demonstrate that the lack of fixation is the optimal condition among all the tested conditions for gene expression analysis, while EtOH fixation proved to be the worst condition.

### Similarity of gene expression profiles

**FIGURE 4:**
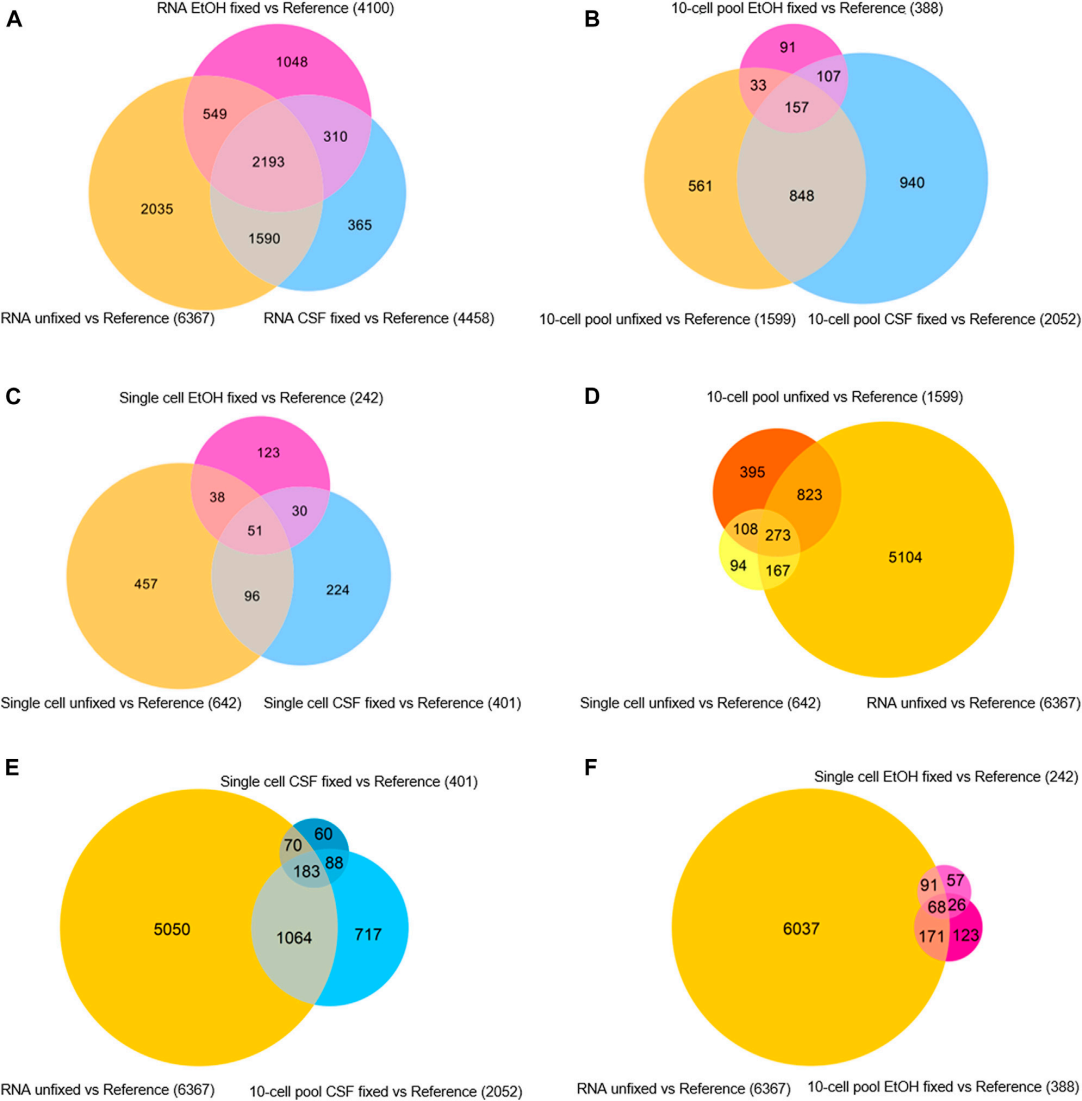
Venn diagrams of differentially expressed genes in **(A)** RNA samples, **(B)** 10-cell pools, **(C)** single cells, **(D)** unfixed RNA, 10-cell pools and single cells, **(E)** unfixed RNA and CSF-fixed 10-cell pools and single cells, **(F)** unfixed RNA and EtOH-fixed 10-cell pools and single cells.

Besides getting insights in the sequencing quality in terms of features detected and genes identified as expressed, we aimed at assessing the level of similarity of gene expression profiles among the samples regardless of their RPKM. To this purpose, gene expression analyses were performed by normalizing the transcriptomic profile of each sample for the XPress Ref Universal Total RNA (Reference) to generate gene expression profiles, intended as a list of upregulated and downregulated genes compared to the Reference. Then, we assessed the level of similarity of expression profiles in function of the fixative condition for each samples type (RNA, 10-cell pool, single cell) by evaluating the generated Venn diagrams (Figure 4).

First, we found that among the RNA samples, the lack of fixation allowed the identification of an increased number of differentially expressed genes compared to the reference (6.367 genes). In addition, 2.193 genes emerged as differentially expressed among the RNA samples regardless of their fixation condition, although CSF-fixed RNA displayed a higher amount of differentially expressed genes shared with the unfixed RNA (3.783 genes) compared to EtOH-fixed RNA (2.742 genes; Figure 4A). Concerning 10-cell pool samples, CSF-fixation provided a higher number of differentially expressed genes (2.052) compared to unfixed (1.599) and EtOH-fixed (388) samples. Overall, 157 genes were shared by all the samples, regardless of their condition, while 1.005 were differentially expressed genes in both CSF-fixed and unfixed 10-cell pools. 264 genes were shared between EtOH-fixed and unfixed 10-cell pools (Figure 4B). Concerning single cells, unfixed samples identified the higher number of differentially expressed genes (642). In CSF- and EtOH-fixed single cells we found respectively 147 and 89 differentially expressed genes in common with unfixed single cells, while 51 genes were shared regardless of their condition (Figure 4C).

Then, we aimed at identifying the fixative condition that allows the achievement of the most reliable OE33 gene expression profile. To this purpose, we assumed the gene expression profile of RNA from OE33 cells unfixed as the most reliable and examined the resulting Venn diagrams (Figures 4D–F). Merging data from unfixed samples highlights that RNA, 10-cell pools and single cells samples share 273 differentially expressed genes (Figure 4D). The list of these genes is reported in Supplementary Table S1. By contrast, gene expression profiles comparison of RNA unfixed vs. CSF-fixed and EtOH-fixed samples resulted 183 (Figure 4E; Supplementary Table S2) and 68 (Figure 4F; Supplementary Table S3) genes, respectively. Interestingly, 10-cell pools CSF-fixed shared with RNA unfixed gene expression profile a higher number of genes (1.247) compared to 10-cell unfixed pools (1.096).

Globally, our findings highlight that transcriptomic profile of unfixed samples are the most reliable, in particular at single cell level. Concerning 10-cell pools, CSF fixation led to the most reliable gene expression profile, compared to the other condition.

## Discussion

Up to now, the development of even more efficient RNA-sequencing techniques and protocols has revolutionized scientific research in the oncology field (Hong et al., 2020; Lei et al., 2021), especially at the resolution of a few down to a single cell. This approach is highly fascinating when applied to rare cells such as CTCs, as their number is often low and requires dedicated workflow for their RNA analysis (Hwang et al., 2018). However, despite the emerging technologies in CTC detection and isolation, this operation remains challenging. One of the most prominent approaches for CTC detection and recovery consists in the DEPArray technology, which proved to be a high-performance ally for the identification and recovery of CTCs and other rare cells by the expression of specific markers (Rossi et al., 2020; Rossi et al.,. 2021b; Gallerani et al., 2021;Rossi et al.,. 2022). Lovero et al. (2022) recently developed a targeted RNA-sequencing assay including a panel of 134 genes involved in metastasis process in DEPArray-isolated CTCs from stage IV breast cancer but no protocols for 3′ transcriptomic analyses through sequencing starting from DEPArray-isolated cells have been identified so far for gene expression purposes. In this study, we describe a protocol for the 3’ RNA-sequencing of OE33 cells isolated by DEPArray NxT platform as single or pooled cells at different conditions of fixation.

The 3’ RNA-sequencing approach is also exploited by the 10X Chromium platform, which is one of the most outstanding emerging technologies for single cell analysis. However, having this technique a capture rate of about 50% (Zheng et al., 2017) and being CTCs underrepresented compared to other cells in the blood (Ferreira et al., 2016), the 10X Chromium technology is not fully applicable to rare cells without a pre-enrichment step (Pauken et al., 2021). At the same time, considered that a high percentage of patients could not have CTCs, the 10X Chromium analysis could fail and be unnecessarily expensive. By contrast, although being a time-consuming procedure, the herein described workflow allows to discriminate positive and negative patients, ensuring to perform downstream analysis on phenotypically investigated CTCs, and to obtain RNA-sequencing data on pure CTCs.

Since one key point in the DEPArray cell recovery procedure is related to the ability of treated cells to be moved within the cartridge, we firstly tested cell quality, and consequently routability (Williamson et al., 2018). At first instance, we decided to include in our test the fixative contained within the CellSave preservative tubes, as they are routinely used for CTC enumeration with the CellSearch instrument. However, the formulation of this preservative is patented and unknown. In literature alcohol-based fixatives, such as methanol and EtOH, are largely used for transcriptomic analyses as their working principle is based on dehydration, thus avoiding chemical modification, and leading to the isolation of high-quality RNA (Alles et al., 2017; Channathodiyil and Houseley, 2021). Hence, EtOH-based fixation was included in our experiments. Based on our results, CSF-fixation negatively affects routability, and seems to have a stronger impact on cell quality. In fact, routability issues could be associated with the presence of cellular debris within the main chamber of the DEPArray cartridge as a consequence of CSF-fixation, making difficult for the instrument to calculate recovery paths. On the other hand, EtOH-fixed cells have a routability rate comparable to unfixed cells.

Next, we aimed at checking whether fixation of OE33 pellet have an impact on the quality of data obtained from the 3’ RNA-sequencing. Based on our results, cell fixation does not negatively impact on RNA quality in terms of mapping efficiency and number of features detected (genes with at least 1 mapping read). Moreover, RNA from fixed cells allows the detection of genes expressed at very low levels (with RPKM value comprised between 1 and 100), accordingly to data from unfixed cells-derived RNA. However, RNA from CSF-fixed cells rather than EtOH had a gene expression profile more similar to RNA from unfixed OE33 cells.

Considered the positive rates of fixed cell routability and the quality of sequencing data from fixed OE33 cells RNA, we decided to proceed with 3’ RNA library preparation starting from unfixed and fixed pooled and single cells, followed by sequencing and bioinformatic analyses through the toolkit for NGS data CLC Genomics Workbench.

Concerning 10-cell pools, our findings highlight that CSF-fixation seems to significantly guarantee a high percentage of mapping reads (67,64%) compared to EtOH (39,82%). In addition, the number of features detected in CSF-fixed pools is in accordance with data from unfixed 10-cell pools, although a fewer amount of less expressed genes was detected. By contrast, EtOH fixation demonstrated a poor efficiency in mapping efficiency and number of features detected, and their sequencing allowed only the detection of genes with a relatively high expression (RPKM>1.000). Again, the CSF-fixed 10-cell pools showed gene expression profiles closest to the unfixed 10-cell pools and RNA, further confirming the limited efficiency of EtOH fixation in this workflow. In addition, we observed that fixed 10-cell pools showed a higher number of genes with RPKM>10.000 compared to unfixed pools. It is known that fixation in some cases may induce expression changes (Kuzmin et al., 2014). In addition, Wang et al. (2021) observed some transcripts had higher expression in fixed cells rather than in unfixed, suggesting their enrichment during library preparation and data normalization.

Lastly, we found a deep discrepancy between results obtained from unfixed and fixed OE33 single cells compared to the other sample types. In fact, while CSF-fixation resulted in acceptable sequencing data in RNA and 10-cell pools, we found that both CSF and EtOH preservatives had equally poor efficiency in number of features detected, and detection of genes with low expression levels (RPKM < 1.000). On the other hand, although the mapping efficiency was comparable to fixed single cells, we observed an increased number of features detected in unfixed single OE33 cells, and improved detection of genes with low expression (100 < RPKM < 1.000). Again, the increased number of genes with RPKM>10.000 may be imputable to library preparation and data normalization (Wang et al., 2021). Further deepen analyses revealed that unfixed single cells shared the major part of their gene expression signature with matched unfixed cells-derived RNA.

Collectively, we found that unfixed cells can be phenotypically analysed and recovered by using DEPArray, and 3’ RNA-sequencing through our workflow provide reliable gene expression results. By contrast, while fixation using EtOH is discouraged, CSF-fixation is suitable in order to get gene expression data in 10-cell pools, but not single cells.

## Data Availability

The datasets presented in this study can be found in online repositories. The names of the repository/repositories and accession number(s) can be found below: GEO, GSE212814.
